# Impact of serum and follicular fluid kisspeptin and estradiol on oocyte maturity and endometrial thickness among unexplained infertile females during ICSI

**DOI:** 10.1371/journal.pone.0239142

**Published:** 2020-10-28

**Authors:** Rehana Rehman, Amara Zafar, Arzina Aziz Ali, Mukhtiar Baig, Faiza Alam

**Affiliations:** 1 Department of Biological and Biomedical Sciences, Aga Khan University, Karachi, Pakistan; 2 Medical College, Aga Khan University, Karachi, Pakistan; 3 Faculty of Medicine, Rabigh, King Abdulaziz University, Jeddah, Saudi Arabia; 4 Department of Physiology, University of Karachi, Karachi, Pakistan; Peking University Third Hospital, CHINA

## Abstract

**Objective:**

To relate serum and follicular fluid (FF) kisspeptin and estradiol levels in different stages of stimulation during Intracytoplasmic Sperm Injection (ICSI) with oocyte maturity and endometrial thickness among unexplained infertile females.

**Methods:**

This cross-sectional study was carried out at the Australian Concept Infertility Medical Centre from March 2017 till March 2018. Fifty unexplained infertile females, booked for ICSI, were included in the study. Serum kisspeptin and estradiol were estimated by Enzyme-Linked Immunosorbent Assay in all four stages; 1: follicular stimulation, 2: ovulation induction, 3: oocyte pickup, and 4: embryo transfer. FF was aspirated during oocyte retrieval (stage 3) for the analysis of KP and estradiol. Pregnancy outcomes were categorized as non-pregnant, preclinical abortion, and clinical pregnancy.

**Results:**

The age of the study subjects was 32.04 ± 2.29 (Mean±SD) years, with mean BMI of 28.51 ± 4.15 (Mean±SD) kg/m^2^. Mean serum kisspeptin and estradiol levels increased in all subjects as the stimulation proceeded stages 1–3; however, the mean dropped after retrieval of the oocytes (stage 4). Out of 27 female subjects who completed the cycle, 17 remained non-pregnant, 4 had preclinical abortion, and 6 acquired clinical pregnancy. The FF kisspeptin concentration was significantly higher than serum concentrations and positively correlated with serum and FF estradiol concentrations. FF-kisspeptin correlated with serum kisspeptin in Stage 3 (r = 0.930, p<0.001), maturity of oocyte (r = 0.511, p = 0.006) and endometrial thickness (r = 0.522, p = 0.005). Kisspeptin in stage 3 was also found to correlate with endometrial thickness (r = 0.527, p = 0.005) and with estradiol (r = 0.624, p = 0.001) independently.

**Conclusion:**

Increase in serum and FF-kisspeptin and estradiol levels from stages 1 to 3, resulted in an optimum endometrial thickness, probability of fertilization of oocytes and chances of clinical pregnancy in Assisted Reproductive Techniques /ICSI cycles of unexplained infertile females.

## Introduction

Regulation of fertility by hypothalamo-pituitary-ovarian (HPO) axis involves the pulsatile release of Gonadotropin-Releasing Hormone (GnRH) from the hypothalamus, stimulating the release of Luteinizing Hormone (LH) and Follicle-Stimulating Hormone (FSH) from the anterior pituitary that in turn acts on the ovaries to control gametogenesis [1]. Kisspeptin (KP) is a neuropeptide that acts via a G-protein coupled receptor and has been found to be the upstream regulator of GnRH release. Hence it plays important roles in female reproduction such as the onset of puberty, ovulation, implantation, placentation and metabolic regulation of fertility [1–3]. The role of KP in puberty onset is supported by the observations that loss of function due to mutations in KP result in the absence of pubertal development, whereas girls with central precocious puberty are found to have higher KP levels [4].

Furthermore, expression of genes encoding KP in ovarian tissue and granulosa-lutein cells as well as on the glandular and luminal epithelial cells of the endometrium, signifies the role of KP in the regulation of reproductive functions [5, 6]. During the ovarian cycle, KP levels increase from the follicular stage to the luteal stage [4]. Estrogen released by the ovaries acts through its receptors present on KP secreting neurons and, in turn, contributes as the negative feedback on KP secretion [6].

Infertility is the inability to conceive after one year of regular unprotected intercourse [1, 7], an attribute of the dysfunction of the HPO, genetic factors, hormonal imbalances and environmental influences [8]. However, in 10–30% of couples seeking infertility treatment, the cause cannot be elucidated, referred to as ‘Unexplained Infertility’ (UI). In UI cases, the tubal patency, anatomy of the uterine cavity, serum progesterone levels in the mid-luteal stage and the semen analysis (male partner) are normal [9]. Treatment options for unexplained infertile females are superovulation and Artificial reproductive techniques (ART), which *In Vitro Fertilization* (IVF) and ICSI [10]

Constituents of FF indicate variations in the ovarian follicle cells in response to the gonadotrophin’s administration, by producing various biochemically active substances. These may affect oocytes’ sustainability and growing capability and are also linked to fertilization outcomes and early post-fertilization development [11]. Literature reveals that follicular fluid (FF) produced by the granulosa cells secrete hormones such as growth hormone, insulin-like growth factor-I, estradiol (E2), and progesterone [11]. Fluctuations in serum and FF-KP levels have been observed during the preovulatory and luteal stages of the reproductive cycle [11–14]. Furthermore, at ovulation induction (OI) during ART, higher serum KP levels are known to be associated with a better pregnancy outcome [6]. Low serum KP levels in UI females [9], and significant correlation of FF-KP with E2 concentrations and follicular maturity in infertile females undergoing ICSI [13], show a link between serum and FF KP concentrations and estradiol with the success of ICSI.

Therefore, we aim to determine the relationship between serum and FF-KP and estradiol concentrations with oocyte maturity and endometrial thickness (determinants of successful conception) among unexplained infertile females during ICSI.

## Material and methods

This cross-sectional study was carried out at the Australian Concept Infertility Medical Center (ACIMC) from March 2017 to March 2018. Ethical approval was obtained from the Institute Review Board of the ACIMC and Aga Khan University, Pakistan (ERC#3331-BBS-ERC-14), and all participants signed written consent.

Women with unexplained infertility (n = 50); duration of infertility more than two years with regular menstrual cycles (21–35 days), normal endocrine profile (normal levels of FSH (<11 IU/L) in early follicular phase (day 2–5), normal Prolactin levels (<20 mg/L) in serum, normal thyroid levels), pelvic ultrasonography with patent fallopian tubes, intact ovaries and uterus, no prior ovarian surgery with normal semen profile of their husband [9, 15] age 24 to 35 years booked for ICSI treatment were included in the study.

Females with uterine fibroids, endometriosis, polycystic ovaries, uterine and cervical lesions, metabolic disorders, and those following the short agonist and antagonist protocol for ICSI, were excluded. Male causes of infertility based on low sperm parameters (sperm count/motility/morphology) according to Kruger’s strict criteria were also omitted [16].

### Treatment protocol

**Fig 1:**
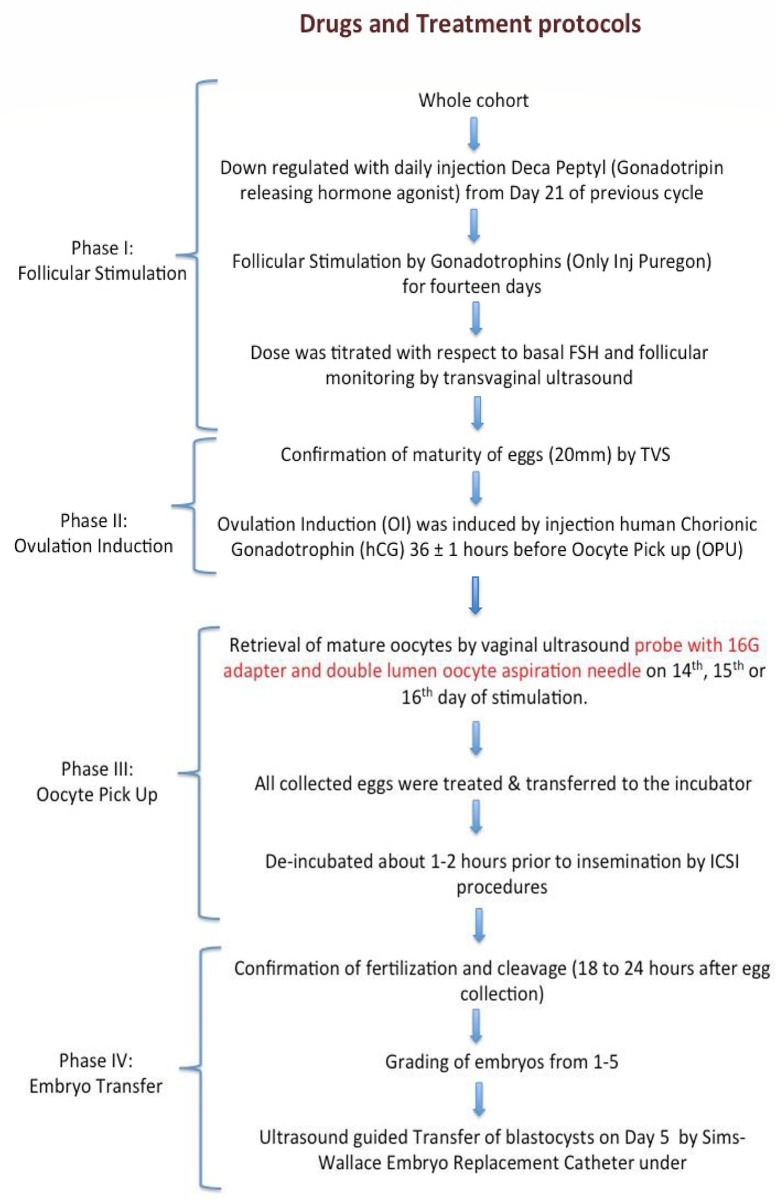
Flow chart of the treatment protocol during the study period. Stage 1: Follicular stimulation (FS) (n = 50). Down-regulation was followed by controlled ovarian stimulation (COS) by gonadotrophin injections (Only Inj Puregon) for fourteen days. The dose was titrated with respect to basal FSH and follicular monitoring by Transvaginal Ultrasound (TVS). Out of the 50 females, 44 females reached the ovarian induction stage (stage 2). Cycle of 4 females was cancelled due to inadequate response to stimulation, whereas two did not opt to continue the treatment. Stage 2: Ovulation induction (OI)(n = 44). On the confirmation of the maturity of at least 3 follicles (measuring18 mm in diameter) by TVS, ovulation was induced by injecting human chorionic gonadotrophin (hCG) 36 ± 1 hour before oocyte pick up (OPU). Maturity of follicles was not confirmed in six females, we could not perform OPU of these females. On the same day, endometrial thickness was measured by a Transvaginal Scan (TVS) in the midsagittal plane by two-dimensional ultrasound with a 7.5 MHz vaginal probe (Hitachi E UB 525; Hitachi, Tokyo Japan) at the thickest endometrial segment [17]. Stage 3: Oocyte Pick Up (OPU) (n = 38). Mature oocytes of females (n = 38) were retrieved 36 hours after hCG injection by vaginal ultrasound probe with 16G adapter and double-lumen oocyte aspiration needle on 14^th^, 15^th^, or 16^th^ day of stimulation. All the collected eggs were treated and then transferred to the incubator for about 1–2 hours prior to insemination by ICSI procedures. During this phase, follicular fluid (FF) was acquired from all follicles (measuring 18 mm or more on ultrasound) from each ovary of the patient [18]. Stage 4: Embryo transfer (ET) (n = 27). After confirmation of fertilization and cleavage (18 to 24 hours after egg collection), embryos were graded from 1–5 [19]. The embryo transfer of 27 females (blastocysts) was done on Day 5 using Sims-Wallace Embryo Replacement Catheter, under ultrasound guidance.

For the downregulation of the hormones, daily injections of Deca-Peptyl (Gonadotropin-releasing hormone agonist) were administered to the patients from Day 21 of the previous cycle. The treatment protocol was carried out in stages (1–4) with sequence of dropouts from the study is given in Fig 1.

### Sample collection

#### Serum samples

The venous blood samples were collected for estimation of serum KP and estradiol at the beginning of stimulation (stage 1), at the time of hCG administration on OI day (stage 2), on OPU (stage 3) and ET days (stage 4). After collecting blood, the serum fraction and the plasma fraction were separated rapidly by centrifuge and frozen at −80°C until assayed. Serum samples were used to detect the concentration of the hormone, using commercially available ELISA kits, following the manufacturer’s protocol. The ELISA kits used were: serum KP (Cat. No: 95611, Glory BioScience, USA) with analytical sensitivity of 10.16 ng/L and intra and inter assay coefficients of variation of less than 10% and 12%, respectively, serum FSH (Kit Cat. No DKO010; DiaMetra) with the inter assay coefficient of variation, <8% and intra assay coefficient of variation <9.7% and serum LH (Kit Cat. No DKO010; Dia Metra) with inter assay coefficient of variation of <7.91%; intra assay coefficient of variation of <9.21%. Serum estradiol was determined using a commercially available kit for Human estradiol (E2) Enzyme Immunoassay Kit (Catalog # 07BC-1111 by MP BioCheck, Inc.).

### Sample of follicular fluid

On the day of oocyte retrieval, two-dimensional mean diameter the follicles, were measured by TVS followed by ultrasound guided aspiration in order to pool FF samples from around one to five follicles of both ovaries, each 18-mm diameter or larger. Samples that appeared blood stained or with oocytes were discarded. For each patient, about 20–40 ml of FF were transferred to sterile conical tubes (BD Falcon, Becton Dickinson, Franklin Lakes, NJ) and centrifuged at 1500 x G for 10 minutes at room temperature. Supernatant was aliquoted in 2ml cryovials and was stored at -80°C refrigerator until further analyzed for hormones [18].

#### Pregnancy outcome

Luteal support of included females (after egg collection) was maintained by progesterone vaginal pessaries (Cyclogest 400 mg) twice a day. We assessed ß-hCG at the end of the treatment approximately 14 days after ET as the outcome marker. TVS was performed 14 days after receiving positive results of ß-hCG to confirm the presence of gestational sac and cardiac activity. Based on ß-hCG and TVS results, women were grouped as: Non-pregnant (NP) if women had ß-hCG 5–25 mIU/ml, Preclinical Abortion (PA) if women had ß-hCG>25 mIU/ml and no fetal cardiac activity on TVS. Women with ß-hCG >25 mIU/ml and evidence of cardiac activity on TVS were grouped as Clinical pregnancy (CP) [20].

### Statistical analysis

All of the clinical and hormonal data were entered and statistically analyzed on SPSS IBM Statistical Package for the Social Sciences (IBM, SPSS version 21;IBM Corp Inc., Armonk, NY, USA). Descriptive analysis of continuous variables was expressed as mean ± standard deviation/error and median (interquartile range). For statistical comparison of variables among stages or groups of patients (NP, CA, CP), analysis of variance (ANOVA) was performed, where p value <0.05 and <0.01 was considered statistically significant and highly significant, respectively. Spearmen correlation coefficient was utilized to detect the effect of hormones on the outcome, p value <0.05 and <0.01 was considered statistically significant and highly significant, respectively.

## Results

The mean age of the study subjects was 32.04 ± 2.29 SD years, with mean BMI of 28.51 ± 4.15 SD kg/m^2^. Clinical characteristics of all participants (n = 50) show mean FSH 7.14 ± 1.55 IU/ml and LH 5.98 ± 1.44 IU/ml. The antral follicle count before COS was 7.91 ± 1.9. The mean dose of puregons (rFSH/day) was 175 ± 24.34 in 44 subjects, OPU of them performed on13 (12–13), median (interquartile range).

### Comparison of outcome groups

The mean number of embryos transferred to 27 subjects were 1.63 ± 0.56. Out of these 27 female subjects, 17 remained NP, 4 had PA, and 6 acquired CP. In these subjects, the number and maturity of oocytes were 7.89 ± 1.85 and 6.89 ± 2.01, respectively, and was highest in the CP group. Table 1 describes comparison of study variables in outcome groups; NP, PA, CP). The number of mature follicles and endometrial thickness were significantly more in CP subjects when compared with NP subjects (p<0.01). The FF- KP and estradiol levels were significantly higher in CP subjects compared to NP subjects (p<0.05) (Table 1).

**Table 1:** Comparison of the descriptive variables between outcome groups.

	Not pregnant n = 17	Preclinical abortion n = 4	Clinical pregnancy n = 6
Age (years)	31.88 ± 2.78	31.25 ± 2.9	33.0 ± 2.9
Body Mass Index (kg/m^2^)	27.82 ± 3.51	29.07 ± 2.24	28.66 ± 7.39
Oocytes (n)	7.35 ± 1.73	7.75 ± 1.5	9.5 ± 1.64*
Metaphase (n)	6.06 ± 1.75	7.25 ± 1.71	9 ± 1.26**
Pronuclei (n)	5.35 ± 1.5	6.5 ± 1.91	6.67 ± 1.21
Endometrial thickness (mm)	6.29 ± 1.79	8.25 ± 2.99	9.5 ± 1.87**
Kisspeptin (Follicular) (pg/ml)	13.46 ± 3.91	16.91 ± 5.31	18.82 ± 4.62*
Estradiol (follicular) (pg/ml)	619320.73 ± 179133.12	484347.72 ± 60164.35	823223.65 ± 188376.92*°

Values are mean ± SD. Results compared by Analysis of Variance; post hoc LSD test was applied

* Significant with Not pregnant at 0.05

** Significant with Not pregnant at 0.01

° Significant with Preclinical abortion at 0.05

°° Significant with Preclinical abortion at 0.01

### Comparison of serum KP and estradiol in various stages of stimulation

**Fig 2:**
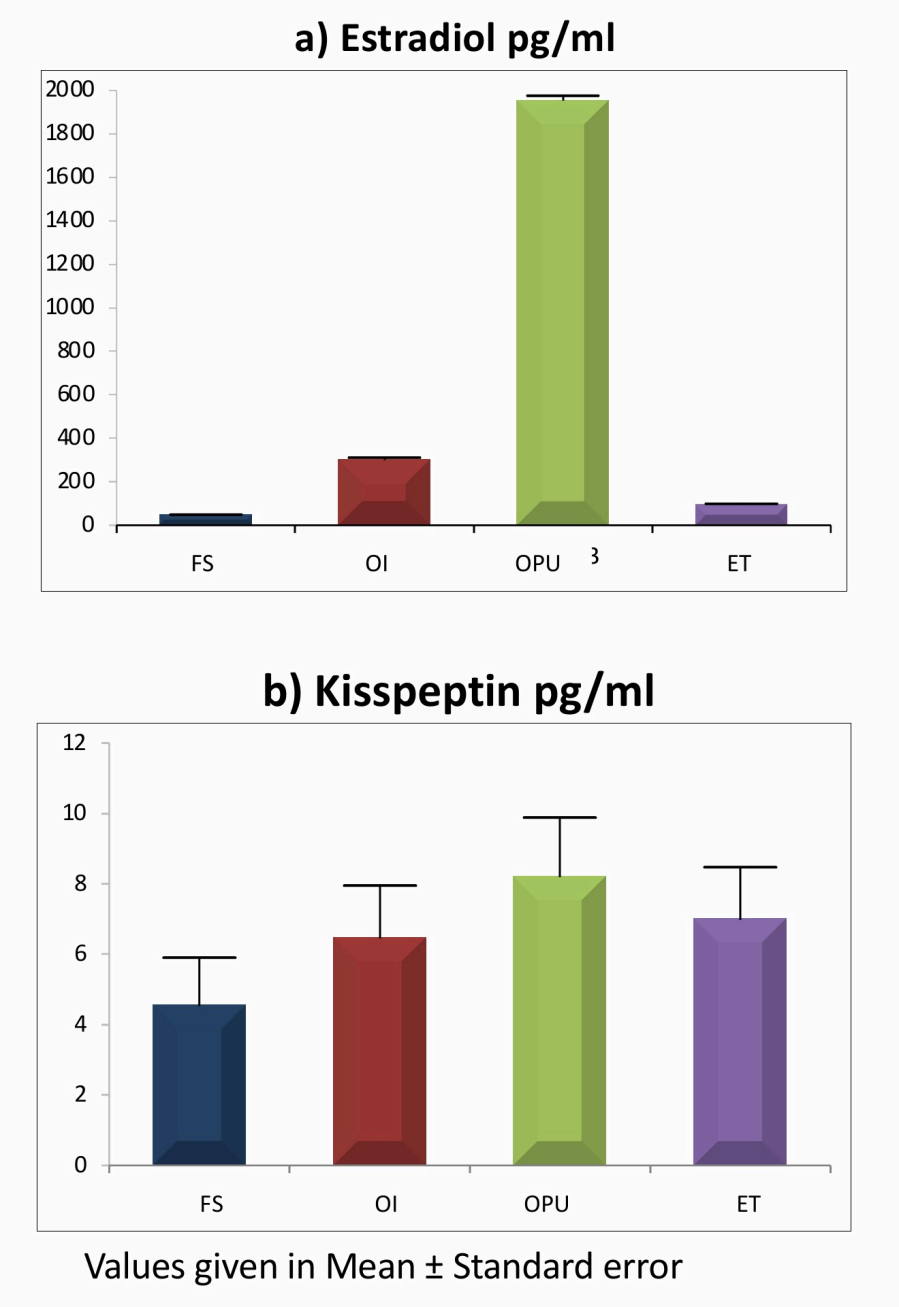
Comparison of estradiol (a) and kisspeptin (b) in all stages of stimulation. *Significant with stage 1 at 0.05 ** Significant with stage 1 at 0.01. Stage 1, FS: Follicular stimulation, Stage 2, OI: Ovulation Induction, Stage 3, OPU: Oocyte pickup, Stage 4, ET: Embryo transfer.

Fig 2 depicts the mean serum KP concentrations were observed to increase within the subjects as the stimulation preceded; however, the mean dropped after retrieval of the oocytes (stage 3). KP showed an increasing trend from stage 1 to stage 3 and was significantly higher in stages 2 & 3 compared to the first stage (p<0.01). A highly significant correlation of KP1 was observed with KP2 (r = 0.845, p<0.001), KP3 (r = 0.833, p<0.001) and KP4 (r = 0.718, p<0.001) among 27 subjects who reached the end of the stimulation successfully.

Estradiol also showed an increasing trend from stage 1 to stage 3, and was significantly higher in the latter two when compared with the first stage (p<0.01). E1 demonstrated a moderately significant correlation with E2 (r = 0.445, p = 0.020), E3 (r = 0.501, p = 0.008) and E4 (r = 0.484, p = 0.011) (Fig 2).

### Comparison of estradiol and KP in outcome groups

**Fig 3:**
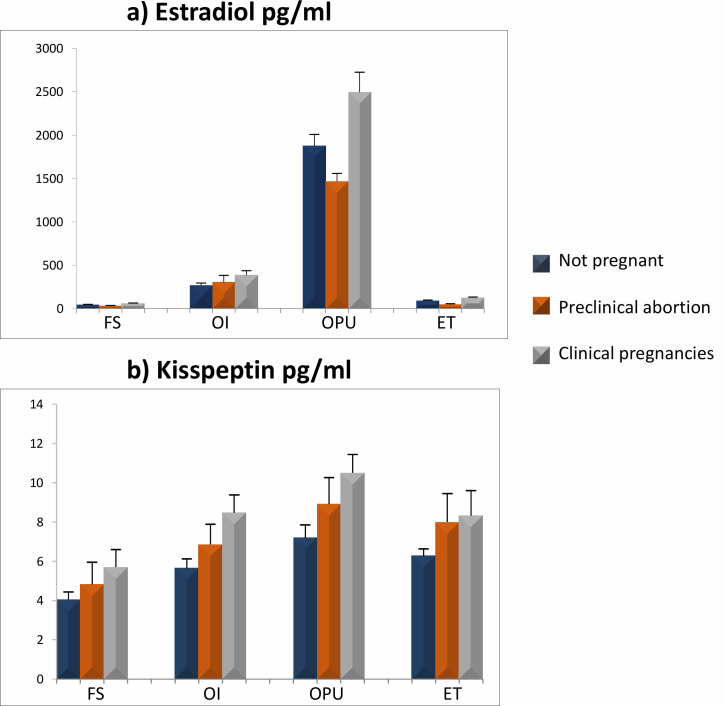
Comparison of estradiol (a) and kisspeptin (b) at various stages of stimulation in all three outcome groups. *Significant with stage 1 at 0.05 ** Significant with stage 1 at 0.01. FS: Follicular stimulation, OI: Ovulation Induction, OPU: Oocyte pickup, ET: Embryo transfer.

Serum E2, E3 and E4 were significantly higher in CP compared to PA subjects (p<0.01). Similarly, serum KP2, KP3 and KP4 levels were significantly higher in CP subjects when compared to NP subjects (p<0.05) (Fig 3).

Serum KP and estradiol levels in NP, PA, and CP subjects showed significantly higher concentrations in OI, OPU and ET (stages 2, 3 and 4) as compared to stage 1 (p<0.01), and the highest levels were found in the 3^rd^ stage. Follicular KP and estradiol levels were highest in the CP females.

### Correlation of serum and FF-KP and estradiol with determinants of pregnancy outcomes

**Fig 4:**
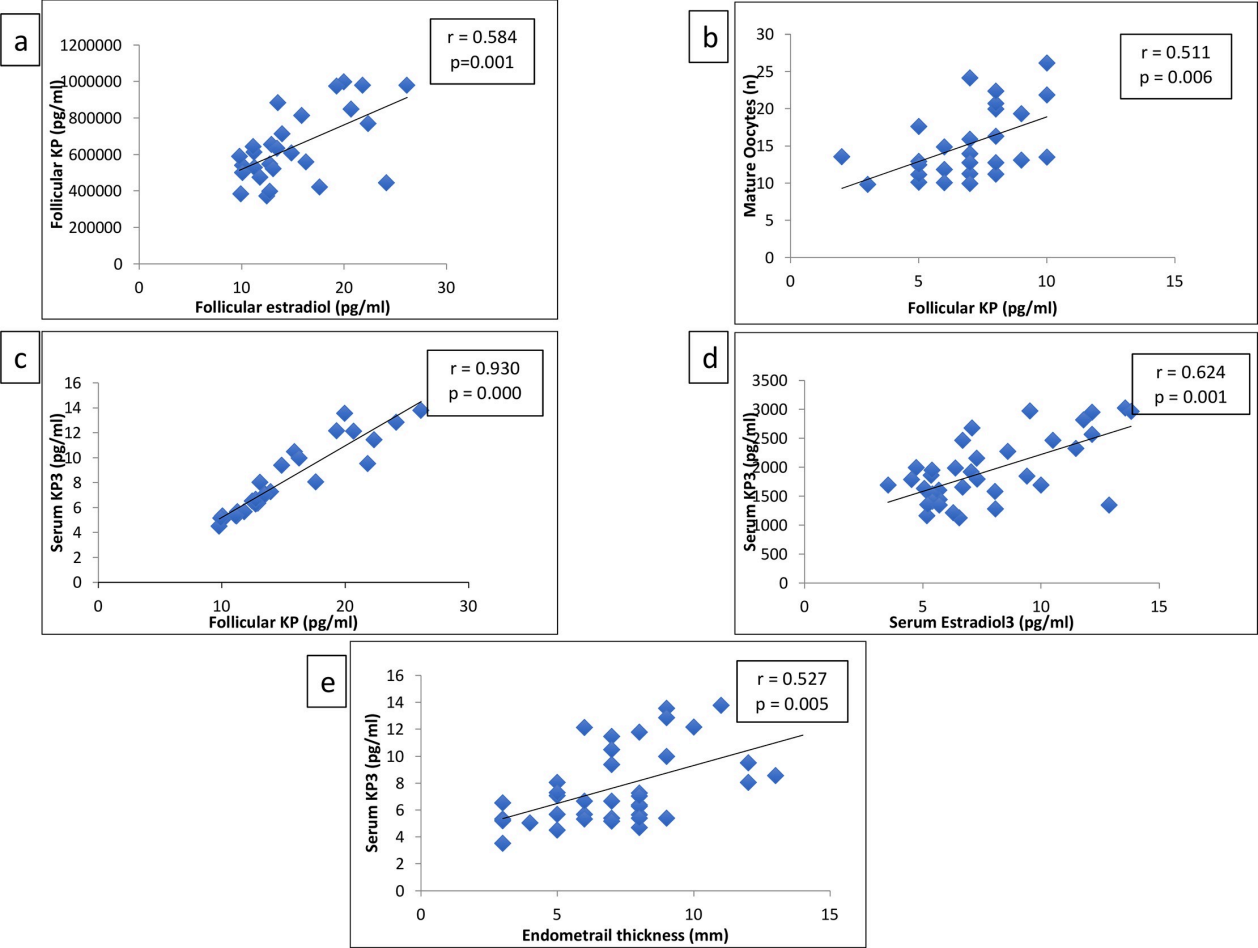
a) Correlation of Follicular estradiol and follicular KP b) Correlation of Follicular KP with number of mature oocytes c) Correlation of Follicular KP with Serum KP3 d) Correlation of Serum KP3 with Serum estradiol e) Correlation of Serum KP3 with endometrial thickness.

A moderate association was found between FF-KP and FF estradiol (r = 0.584, p = 0.001) (Fig 4A). The FF- KP was observed to correlate with the oocyte maturity (r = 0.511, p = 0.006) (Fig 4B) and KP3 (r = 0.930, p = 0.000) (Fig 4C). Furthermore, KP3 was positively associated with E3 (r = 0.624, p = 0.001) (Fig 4D) and endometrial thickness (r = 0.527, p = 0.005) (Fig 4E).

## Discussion

Kisspeptin is known to control the pulsatile surge of GnRH release, ovulation, implantation, placentation, and metabolic regulation of fertility [2]. Wang and Moenter, (2020) suggested that arcuate and anteroventral periventricular KP neurons perform definite functions in facilitating the response to systemic variations in estradiol levels, standardizing cyclicity and LH rise [21]. Our study showed that ART was not successful in females with lower serum and FF- KP levels. The results are in accordance with KP's role in eliciting the LH surge required for oocyte maturation and ovulation. Jamil et al. (2017) reported a significantly higher KP, LH, basal and peak estradiol after ICSI treatment, in a positive pregnancy subjects compared to the negative pregnancy subjects [6]. Besides, endometrial thickness, oocyte numbers (matured and fertilized), embryo quality, and potential for developing to the blastocyst stage were notably better in the positive pregnancy group [6]. Moreover, they reported a positive correlation between raised KP and positive pregnancy outcome, and a borderline correlation of KP with the endometrial thickness [6]. A recent study reported no significant difference in serum and FF KP levels in patients undergoing in vitro fertilization [2]. However, they observed significant increase in serum KP levels in patients treated with ovarian hyperstimulation. Interestingly, in contrast to our results, there was no significant difference in KP levels between pregnancy negative and positive subjects either in serum or FF after ovarian hyperstimulation [22]. Furthermore, no correlation was found between FF and serum KP in all patients and pregnancy positive patients. They didn’t find any significant correlation between estradiol and FF and serum KP levels [22]. They also reported that by employing multivariate stepwise linear regression, the number of oocytes was considerably affected by the hyperstimulation-induced increase in serum KP. Few studies have reported improvement in oocyte maturation after the administration of synthetic KP that caused a controlled LH rise in women at high risk of ovarian hyperstimulation syndrome (OHSS) [23, 24].

Our study observed that serum KP levels increased from FS to OPU stages of ART, followed by a decrease in ET stage. The findings are consistent with the results of another study [13]. A Saudi study showed a significant increase in serum KP and E2 levels from the early follicular to the preovulatory and the luteal stage among normal menstruating female medical students [14]. In contrast to our results, they documented an insignificant correlation between KP and E2 in all three stages. At present, we don’t have any rationale for this difference. Recently, Yu et al. (2019) reported role of KP as a placental marker by finding correlation of KP with pregnancy outcomes, and noticed that women with biochemical pregnancy loss had lower KP levels [25]. Mumtaz et al. (2017) also reported higher KP levels among the CP group as compared to NP and PA groups [9]. However, in their study, blood samples were taken from study participants only before initiation of stimulation for ICSI.

We found that FF KP concentration was higher than serum KP, similar to Taniguchi et al., confirming the presence of KP receptors in the granulosa cells and secretion by the ovary [13]. They also found that increase in KP correlated with E2 production in all stages of the ovarian cycle, suggesting that higher KP and E2 levels are supportive of attaining pregnancy. Emerging evidence on FF- KP levels and its relationship with the female reproductive system reflects the importance of intraovarian KP in follicular development, oocyte maturation, and ovulation. Decreased concentration of follicular KP can adversely affect ovarian function, female reproduction, and fertility [26]. It has been observed that granulosa cells (GC) are one of the major sites for KP synthesis in rats, as Kiss1mRNA expression was found to be significantly higher in the GCs compared with the theca cells and other ovarian cells [26]. In our study, KP levels in FF were higher than maximum serum KP levels in women, which is similar to a study that also observed higher FF KP concentration than serum levels [13].

It has been observed that the high serum (peak) E2 around the time of ovulation impacts the number, and maturity of oocytes and the thickness of endometrial lining [23, 24]. Its measurement on OI day for predicting success in the treatment of ICSI has also been recognized [19]. Our results showed maximum serum E2 levels at the time of OPU (stage 3) in CP subjects. Higher E2 levels observed on the day of OI were maintained in luteal phase of conception cycles in another study [27].

The serum KP concentration, the number of fertilized oocytes, and the endometrial thickness were found to be more significant factors contributing in unexplained infertile women in becoming pregnant as compared to those who did not conceive. Our results are similar to a study that included infertile females with different infertility causes [28]. The result of a clinical trial using KP-54 as an inductor of oocyte maturation (in IVF treatment) showed that there is a dose-dependent increase in the number of mature oocytes, even though the rate of oocyte maturation remained similar across the doses [29]. Another clinical trial study indicated that synthetic KP-54 is a promising choice for oocyte maturation in IVF treatment, especially in patients at high risk of OHSS [23]. Till now, several trials used synthetic KP-54 for oocyte maturity, and they suggest its suitability for oocyte maturation induction, specifically in OHSS. Nevertheless, there is a need to compare KP with current oocyte maturation methods [30].

In our study, the number of oocytes and the endometrial thickness was the highest in those attaining CP, consistent with the highest serum and FF- KP levels in the similar women. This suggests the positive effect of high levels of FF-KP on the number of oocytes, their fertilization, endometrial thickness, favorable implantation of an embryo, and the success of ART in achieving pregnancy. Literature supports the efficacy of Kisspeptin-54 in triggering the maturation of oocytes specially in high risk cases of OHSS [23]. We found a significant positive association between FF- KP level and estradiol concentration, furthermore, FF-KP was also observed to correlate with the maturity of oocytes. These results agree with a previous study that reported the positive correlation between FF-KP with serum and FF estradiol levels and the number of mature eggs [13]. These outcomes suggest a link between FF KP concentration and egg maturity.

Our results indicate that KP plays a role in oocyte maturity, and its decreased levels in serum and FF not only affect the normal fertility process but also have the potential to influence IVF/ICSI outcomes. Further studies are required to understand the role of KP that could direct its employment as a biomarker in treating infertile subjects and explore its utility in COS [4].

### Limitations

The present study has a few limitations, such as small sample size and the cross-sectional nature of the study. Furthermore, the genetic and immunological causes of infertility could not be investigated in our study. We need to design longitudinal studies with larger sample size. The generalizability of the results is not possible since it was a unicentric study. We tried to understand the relationship between serum and FF- KP and estradiol levels with the determinants of pregnancy outcome.

## Conclusions

In ICSI cycles, serum KP and increased from the initiation of stimulation to egg collection (from stages 1 to 3), with a higher concentration depicted in FF. The stimulated granulosa increased the production of estradiol, which was reflected in both serum and FF concentrations, during these stages. Estradiol production reached its maximum in the OI phase. A strong positive correlation of KP3 and E3 with the determinants of successful conception; number of fertilized oocytes and endometrial thickness emphasize on the role of KP and estradiol in successful conceptions. Thus increase in serum and FF of KP and estradiol levels assisted in acquiring an optimum endometrial thickness, and fertilization of oocytes with more chances of clinical pregnancy in ART /ICSI cycles of UI females.

We recommend further clinical trials with more UI females to determine the impact of serum and FF -KP and estradiol on UI females’ outcome measures during ICSI. This will help us understand the therapeutic options for KP in treating infertility, especially the unexplained cases.

## Supporting information

S1 Table Comparison of kisspeptin and estradiol at the time of follicular stimulation with other phases of stimulation. (DOCX)

S2 Table. Comparison of kisspeptin and estradiol in outcome groups in various phases of stimulation (DOCX)
