# Supplementary material for: Impact of serum and follicular fluid kisspeptin and estradiol on oocyte maturity and endometrial thickness among unexplained infertile females during ICSI
Source: PLoS One. 2020 Oct 28;15(10):e0239142. doi: 10.1371/journal.pone.0239142 (PMC7593084; doi:10.1371/journal.pone.0239142)
Supplement: S1 Table — (DOCX) [file pone.0239142.s001.docx]

**S1 Table:** Comparison of Kisspeptin and Estradiol at the time of follicular stimulation with other phases of Stimulation

|  |  | Stage 1 | Stage 2 | Stage 3 | Stage 4 |
| --- | --- | --- | --- | --- | --- |
| Kisspeptin (pg/ml)  (n=27) | Mean ± SD | 4.53 ± 1.92 | 6.45 ± 2.3** | 8.19 ± 2.91** | 6.98 ± 2.27** |
|  | *rho value* |  | 0.845** | 0.833** | 0.718** |
| Estradiol (pg/ml) n=27) | Mean ± SD | 46.28 ± 18.68 | 300.47 ± 125.7** | 1953.44 ± 596.29** | 92.89 ± 40.38** |
|  | *rho value* |  | 0.445* | 0.501** | 0.484* |

* Significant with phase 1 at 0.05

** Significant with phase 1 at 0.01
