# Supplementary material for: Impact of serum and follicular fluid kisspeptin and estradiol on oocyte maturity and endometrial thickness among unexplained infertile females during ICSI
Source: PLoS One. 2020 Oct 28;15(10):e0239142. doi: 10.1371/journal.pone.0239142 (PMC7593084; doi:10.1371/journal.pone.0239142)
Supplement: S2 Table. Comparison of kisspeptin and estradiol in outcome groups in various phases of stimulation — (DOCX) [file pone.0239142.s002.docx]

**S2 Table:** Comparison of Kisspeptin and estradiol in outcome groups in various phases of stimulation.

| Outcome  Groups | Serum levels | | | | | Follicular Fluid levels |
| --- | --- | --- | --- | --- | --- | --- |
|  | Variables | Stage 1 | Stage 2 | Stage 3 | Stage 4 |  |
| Not pregnant n=17 | Kisspeptin pg./ml  (Mean ± SD) | 4.05 ± 1.64 | 5.64 ± 2** | 7.2 ± 2.75** | 6.27 ± 1.49** | 13.46 ± 3.91pg./ml |
|  | Estradiol pg./ml  (Mean ± SD) | 44.97 ± 17.89 | 269.07 ± 109.05** | 1876.73 ± 542.83** | 92.13 ± 37.99** | 619320.73±179133.12  pg./ml |
| Preclinical abortion n=4 | Kisspeptin pg./ml  (Mean ± SD) | 4.82 ± 2.29 | 6.86 ± 2.08** | 8.92 ± 2.71** | 7.99 ± 2.92* | 16.91±5.31  pg./ml |
|  | Estradiol pg./ml  (Mean ± SD) | 31.69 ± 11.95 | 305.58 ± 158.11* | 1467.72 ± 182.32** | 48.39 ± 20.08 | 484347.72± 60164.35  pg./ml |
| Clinical pregnancies n=6 | Kisspeptin pg./ml | 5.7 ± 2.21 | 8.48 ± 2.21** | 10.5 ± 2.32** | 8.32 ± 3.15* | 18.82 ± 4.62 pg./ml |
|  | Estradiol pg./ml | 59.69 ± 17.66 | 386.02 ± 130.18** | 2494.62 ± 570.84** | 124.73 ± 28.54** | 823223.65 ± 188.376 |

*Significant with phase 1 at 0.05

** Significant with phase 1 at 0.01

1: In Follicular stimulation

2: In Ovulation Induction

3: In Oocyte pickup

4: At Embryo transfer
